# *Sarcocystis camelicanis* increases interleukin (IL)-6 expression in one-humped camels (*Camelus dromedarius*) from Riyadh and Al Qassim, Saudi Arabia

**DOI:** 10.1042/BSR20203140

**Published:** 2021-01-19

**Authors:** Dina M. Metwally, Tahani T. Al-Otaibi, Abdelhabib Semlali, Reem A. Alajmi

**Affiliations:** 1 Department of Zoology, College of Science, King Saud University, Riyadh, KSA; 2 Department of Parasitology, Faculty of Veterinary Medicine, Zagazig University, Zagazig, Egypt; 3 Department of Biology, Al-Nairiyah University College, Hafr Al-Batin University, Saudi Arabia; 4 Groupe de Recherche en Écologie Buccale, Faculté de Médecine Dentaire, Université Laval, Québec, Canada

**Keywords:** Camelus dromedarius, IL-6, Sarcocystis camelicanis

## Abstract

*Sarcocystis* spp. are intracellular protozoan parasites with an intermediate-definitive host life cycle based on a prey–predator relationship. Sarcocystis infection is common among different vertebrates including humans. The pathogenicity of *Sarcocystis* spp. is of varied significance including a possible lethal effect for the host. The goal of the present study was to investigate the inflammatory activity of *Sarcocystis* spp. in different organs of naturally infected camels. The tongue, esophagus, heart, diaphragm, and skeletal muscles were collected from 50 camels, and the tissues assessed for the presence of *Sarcocystis* spp. by macroscopic examination, light microscopy, and transmission electron microscopy (TEM). Moreover, expression of the interleukin (IL)-6 was analyzed using reverse transcriptase quantitative polymerase chain reaction (qPCR). Microscopic *Sarcocystis* spp. cysts were found in camels. TEM identified the cysts as *Sarcocystis camelicanis* (*S. camelicanis*). *Sarcocystis* infection increased inflammation by stimulation of IL-6 expression in different organs of the camels, particularly in those from the Al-Qassim region.

## Introduction

*Sarcocystis* species are apicomplexan protozoa in the class Conoidosida and the subclass Coccidia [1] and are intracellular parasites that infect mammals, reptiles, and birds [2,3]. Sarcocystis infection may cause severe diseases in their intermediate hosts sheep, goats, cattle, and pigs [4]. Clinical features of acute sarcocystosis include weakness, loss of weight, anemia, fever, edema, diarrhea, hemorrhage, muscle twitching, muscle atrophy, hair loss, increased salivation, and decreased lactation [5]. Severe sarcocystosis can cause hepatitis, encephalitis, encephalomyelitis [5,6], and even abortion in infected pregnant females [7]. Therefore, sarcocystosis is considered a life-threatening disease for many animals of economic importance [8]. *Sarcocystis* spp. are immunogenic in intermediate hosts; information on cellular and humoral immune responses is available only from responses against antigens derived from bradyzoites [9]. Immune cell activation during sarcocystosis is similar to the activation pattern during an intracellular parasite infection, where immune cells such as lymphocytes and macrophages are activated and invade the viscera and muscles [9]. Mononuclear cell infiltration starts 3 weeks after infection and may continue for some time after the parasite is no longer noticeable in visceral tissues. Similarly, lymphocytes from peripheral circulation demonstrate a blastogenic reaction when activated by antigen-specific *Sarcocystis* spp., regardless of whether these cellular events contribute to the restoration of the host from the disease [4]. Immune animals that resist fatal infections display cell-mediated immune response to resist sarcocysts, including cytotoxic antibodies or metabolites that are known to damage second generation extracellular merozoites [10].

Interleukin (IL)-6 is a cytokine with pleiotropic and redundant actions that aids host defense in response to acute environmental stress. However, unrestrained persistent IL-6 production has been confirmed to present a pathological role in many autoimmune and chronic inflammatory diseases. Influencing IL-6 is therefore a logical approach to treat these diseases [11]. At the beginning stage of infectious inflammation, IL-6 is produced by monocytes and macrophages directly after the stimulus of Toll-like receptors (TLRs) with definite pathogen-associated molecular patterns [12]. In non-infectious inflammations, as burns or traumatic injuries, damage-associated molecular patterns from damaged cells promote TLRs to yield IL-6 [13]. This acute IL-6 expression performs a crucial role in host defense by promoting proliferation of immune cells. Transitionary synthesis of IL-6 results in a rapid contribution to the host defense against infection and injury and simultaneously provides a warning signal by activating a broad spectrum of biological events. Once the source of stress is eliminated from the host, IL-6-mediated activation of the signal transduction cascade is dampened by negatively regulatory systems in combination with the normalization of serum IL-6 and C-Reactive Protein (CRP) levels. However, uncontrollable persistent IL-6 production has been associated in the development of various autoimmune and chronic inflammatory diseases and even cancers [14–17]. Many studies have shown that IL-6 has anti-parasite protective responses against some tissue parasites as *Trypanosoma cruzi* [18,19], *Toxoplasma gondii* [20], and *Leishmania major* [21].

In Northern Africa, the Middle East, Central Asia, and China, camel meat is preferred due to low cholesterol/fat content. Sarcocystosis has been reported more frequently in areas where camels are reared in the presence of rural dogs [21,22]. Until now, two *Sarcocystis* spp., *Sarcocystis ippeni* and *Sarcocystis cameli*, have been identified in camels [23]. The infected tissues may show no degenerative or inflammatory responses. However, necrosis and an inflammatory response in muscle fibers have been observed in some of the infected tissues and are accompanied by infiltration of polymorphonuclear cells, lymphocytes, macrophages, eosinophils, and fibroblasts [24,22]. The objective of the present study was to analyze the mRNA expression of cytokine IL-6 in camels naturally infected with *Sarcocystis* spp. from two different regions in Saudi Arabia to disclose the effect of IL-6 on the disease development.

## Materials and methods

### Sample collection

Tissue samples were collected by veterinarians during post-mortem inspections of animals slaughtered at the West Abattoir in Riyadh and Onaizah Modern Slaughterhouse in Al Qassim, Saudi Arabia between February and October, 2018. Official approval of the use of tissues for research purposes was obtained from the university as well as the abattoir veterinarians. Tissue samples were isolated from 50 camels (25 camels from Riyadh and 25 camels from Al Qassim). The entire tongue, heart, skeletal muscle, diaphragm, and esophagus were collected from each animal and individually stored in sealed plastic bags. The tissues samples (250 samples, 125 samples from five organs from each region) were then transported to the laboratory in boxes containing ice packs.

### Macroscopic analysis

Tissues were analyzed macroscopically the same day they were collected. The tongue and heart were transversely sectioned into three sections to reveal macroscopic cysts. The entire esophagus was longitudinally sectioned to expose the lumen, and the internal and external walls were analyzed macroscopically [23].

### Microscopic analysis

Fresh tissues were microscopically analyzed for cysts by squash preparation [23]. Approximately 5 mm thick tissue fragments were firmly squashed between two slides and examined under (ECLPSE NI-4, Nikon, Japan) microscope at 40× and 100× magnification.

### Tissue digestion for sarcocyst detection

Approximately 20 g of each tissue was minced and digested for 40 min at 37°C in 100 ml digestion medium with 1.3 g pepsin, 3.5 ml HCl, and 2.5 g NaCl in 500 ml of distilled water [4]. After digestion, the mixture was centrifuged for 3 min at 3500× ***g*** in a (UNIVERSAL 320 R, Hettich, Germany), and then the sediment was stained with Giemsa and examined under a (ECLPSE NI-4) microscope at 400× magnification [25].

### Transmission electron microscopy (TEM)

Six *Sarcocystis* spp. cysts implanted in the tissues were gathered from organs, fixed in 0.1 M sodium cacodylate buffer (pH 7.4), supplemented with 3% glutaraldehyde solution, for 4 h at 4°C, and then stored at 4°C until processing. After fixation, the samples were washed in 0.1 M sodium cacodylate buffer, fixed with 2% osmium tetroxide for 24 h, and re-washed four to five times in the buffer (10–15 min each) [26]. Samples were serially dehydrated in increasing concentrations of acetone (30%, 40%, 50%, 70%, 90%, and 100%), and blocked with buffer supplemented with 1% phosphotungstic acid and 1% uranyl acetate. Next, the 100% acetone solution was replaced with Polybed resin, followed by paraffin embedding and polymerization in an oven at 60°C [27]. Moderately thin sections were made to observe the *Sarcocystis* spp. cysts by microscopy (CX31, Olympus Corporation, Japan). Ultrathin sections were stained with uranyl acetate and lead citrate and then examined using a JEM-1400 transmission electron microscope (JEOL, Japan) at 80 kV.

### Total RNA isolation

Frozen tissues samples at −20°C (preserved in RNAlater™ Stabilization Solution Catalog number: AM7021) of cardiac muscle (Group 1), tongue (Group 2), diaphragm (Group 3), and esophagus (Group 4); each group had three infected samples and three non-infected samples with 48 total samples. Samples (20 mg) of each tissue were homogenized using Medic Tools homogenizer machine (gentle MACS Dissociator, Germany). Total RNA was isolated from infected and non-infected tissue samples using GeneAll Ribospin II RNA Extraction Kit (GeneAll Biotechnology, Seoul, Korea). The extracted RNA concentration and purity were measured by Biospectrometer (Eppendorf, Germany).

### Complementary DNA (cDNA) synthesis

cDNA was synthesized by HyperScript First Strand Synthesis Kit (GeneAll Biotechnology, Seoul, Korea). Briefly, 1 µg isolated RNA with 10 µl Master mix was applied to the Mastercycler (Eppendorf) for the Polymerase Chain Reaction (PCR). The conditions for preparing the cDNA were 5 min at 65°C, 1 h 30 min at 55°C, and 5 min at 85°C. The cDNA prepared was stored at −20°C.

### Quantitative PCR (qPCR)

Cytokine expression levels were assessed via a previously reported qPCR protocol [28–30], using Power SYBR™ Green PCR Master Mix and a 7500 Real-Time PCR System (Applied Biosystems, Darmstadt, Germany). Specific primers for IL-6 (Forward, 5′-GGAACGAAAGAGAGCTCCATC-3′; Reverse, 5′- CTCATCATTCTTCTCACATATCTCC-3′) and control GADPH (Forward, 5′-GGTATCGTCGAAGGACTCATGAC-3′; Reverse:5′-ATGCCAGTGAGCTTCCCGTTCAGC-3′) were used [31] as IL-6 is relative marker for *Sarcocystis* spp. infections.

Each reaction volume (25 µl) included 12.5 µl SYBR Green, 0.5 µl primer (Forward and Reverse), 7 µl distilled water, and 5 µl of the cDNA. The conditions of RT-qPCR for the hold, PCR, and melt curve stages were 2 min at 50°C and 5 min at 95°C; 30 s at 95°C, 45 s at 72°C, and 15 min at 95°C; and 30 s at 95°C, 45 s at 60°C, and 15 min at 95°C, respectively. The relative mRNA transcripts amount were measured by their Cycle Threshold (CT) values using Applied Biosystems software. The results were analyzed by the Livak relative expression method [32].

### Statistical analysis

Experiments regarding the inflammatory activity of Sarcocystis were repeated more than three times independently and in triplicate. The data were analyzed by using SPSS statistical software. All data were analyzed as a completely randomized design using independent sample *t*-test to compare between data of two experimental groups. The results were presented as fold changes mean ± standard error of mean.

## Results

### Natural infection prevalence

The tissues were only infected by *S. camelicanis* and cleared with other potential bacterial or viral infections. The prevalence of Sarcocystis cysts varied from one camel organ to another, and from one region to another. In general, the diaphragm and esophagus had the highest infection levels of all organs examined (Riyadh diaphragm, 44%; Riyadh esophagus, 36%; Al-Qassim diaphragm, 40%; Al-Qassim esophagus, 32%). The skeletal muscle had the lowest infection rate (Riyadh, 16%; Al Qassim, 13%) (Table 1). Tissues were considered as non-infected if they were negative for the presence of any cysts following examination in triplicate by squash preparation and once by digestion.

**Table 1:** Prevalence and organ distribution of *S. camelicanis* cysts in camels from Riyadh and Al-Qassim regions

Tissues	Regions			
	Riyadh		Al-Qassim	
	No. of examined	No. of infected (%)	No. of examined	No. of infected (%)
**Tongue**	25	7 (28%)	25	5 (20%)
**Heart**	25	7 (28%)	25	5 (20%)
**Skeletal muscle**	25	4 (16%)	25	3 (12%)
**Diaphragm**	25	11 (44%)	25	10 (40%)
**Esophagus**	25	9 (36%)	25	8 (32%)

### Morphological characteristics of the cysts

**Figure 1:**
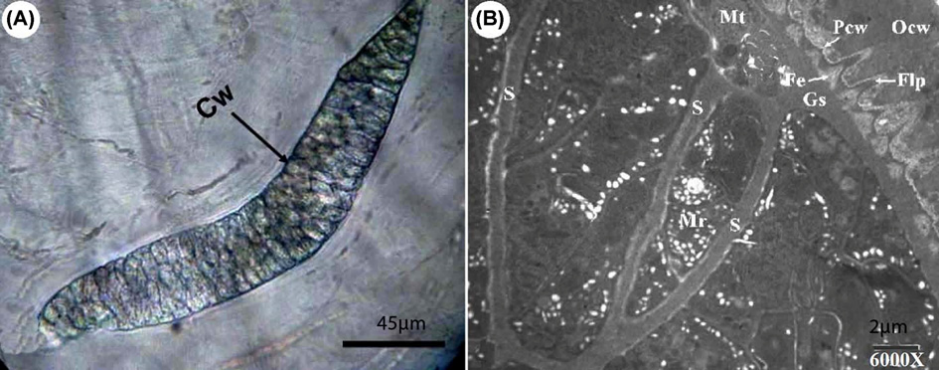
Morphology of a sarcocyst of *S. camelicanis* (**A**) Squash technique in camel esophagus: cyst wall (Cw) (100 µm); (**B**) General view of thick walled sarcocysts of *S. camelicanis* showing Cw, finger like processes (Flp), fibrillar elements (Fe), Primary cyst wall (Pcw), ground substance (Gs), septae (S), metrocytes (Mt), and merozoites (Mr) (×6000).

An investigation of muscle samples including cardiac muscle, tongue, diaphragm, esophagus, and skeletal muscles obtained from 50 slaughtered camels revealed only microscopic thick-wall cysts of *S. camelicanis*; macroscopic cysts were not observed in the present study. The pepsin-hydrochloric acid digestion technique detected a higher number of cysts than detected by the tissue squash method. No bradyzoites were detected by digestion technique. The cysts were fusiform or spindle-shaped, ran parallel to the muscle fibers, and measured 150–450 µm in length and 64–139.8 µm in width (mean 300 × 101.9 µm^2^) (Figure 1A). The populations of microcysts in camel tissues from Al Qassim were higher than those from Riyadh. An ultrastructure analysis (Figure 1B) of the cyst wall revealed that *S. camelicanis* has an outer cyst wall connected to the primary cyst wall (Pcw), ground substance (Gs), metrocytes, and bradyzoites.

The Pcw is a thick dense layer adjacent to Gs. Fibrillary elements (Fe) originating from Gs 0.60 µm below the Pcw aggregate toward the Pcw and embed into this to form finger-like protrusions (Flp). The Flp measure between 2.15–2.91 µm (mean 2.53 µm) in length and 0.52–0.60 µm (mean 0.56 µm) in breadth. Each Flp carries characteristic multiple numerous knob-like structures (Kls) that are spherical in shape with a 0.10–0.14 µm (mean 0.12 µm) diameter; the Kls number varies from 19 to 25 on each protrusion. The distance between each Flp was observed to be 0.71–0.89 µm (mean 0.80 µm) and was usually surrounded by numerous dispersed host cell mitochondria. Gs was found 1.25–1.60 µm (mean 1.42 µm) below the Pcw and between the Pcw and the metrocytes. The Gs appeared as a homogeneous substance and extended to the interior of the cyst by the septa, which separates the cyst into a number of compartments enclosing the metrocytes, merozoites, and other structures.

### Inflammatory activity of *S. camelicanis*

**Figure 2:**
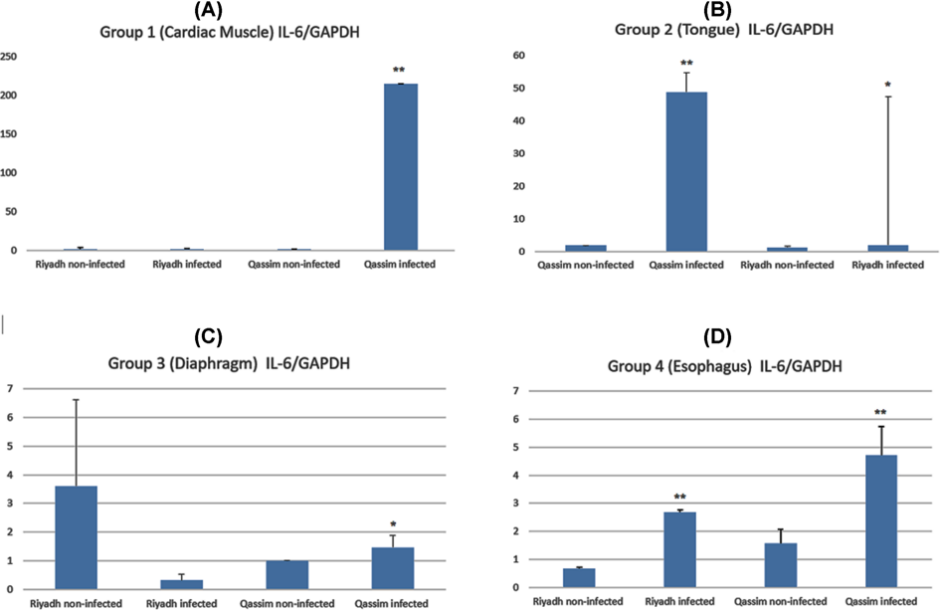
Relative cytokine mRNA expression levels after infection with *S. camelicanis* sarcocysts in different tissues of camel (**A**) Cardiac muscle, (**B**) tongue, (**C**) diaphragm, and (**D**) esophagus. * No significant difference in IL-6 expression in infected and non-infected camels. ** Significant difference in IL-6 expression in infected and non-infected camels.

To investigate the relation between gene expression of inflammatory cytokines and *S. camelicanis* cyst infection in different organs of camels, we used RT-qPCR to compare the gene expression of inflammatory cytokines in infected tissues from cardiac muscles, diaphragms, tongues, and esophagi with that in non-infected tissues. The variation of mRNA levels of IL-6 is summarized in Table 2 and Figure 2. The skeletal muscles were not investigated due to the low number of cysts isolated from these muscles.

**Table 2:** Expression of IL-6 with *S. camelicanis* infection in different organs compared with non-infected control

Organs	Groups	IL-6/GAPDH	SEM
**Cardiac muscle**	Riyadh (non-infected)	1.56	1.30
	Riyadh (infected)	1.52	0.5
	Al Qassim (non-infected)	0.97	0.01
	Al Qassim (infected)	214.54	0.12
**Tongue**	Riyadh (non-infected)	1.19	0.03
	Riyadh (infected)	1.92	5.88
	Al Qassim (non-infected)	1.84	0.49
	Al Qassim (infected)	48.70	45.40
**Diaphragm**	Riyadh (non-infected)	3.60	3.02
	Riyadh (infected)	0.31	0.22
	Al Qassim (non-infected)	1.00	0.01
	Al Qassim (infected)	1.46	0.43
**Esophagus**	Riyadh (non-infected)	0.66	0.03
	Riyadh (infected)	2.67	0.07
	Al Qassim (non-infected)	1.57	0.35
	Al Qassim (infected)	4.71	0.73

mRNA expression of the proinflammatory cytokine Il-6 increased approximately 215-fold in cardiac muscle tissues of infected camels from Al Qassim compared with that in similar tissues of healthy camels (Figure 2A). No significant difference in IL-6 expression was observed in either infected or healthy camels from Riyadh. In the tongue, the infection by *S. camelicanis* increased the IL-6 expression approximately 48-fold in Al Qassim but only 0.7-fold in Riyadh (Figure 2B). No significant difference was observed in IL-6 expression in the diaphragm tissue of infected animals from Riyadh compared with that in the diaphragm tissue of uninfected animals (Figure 2C).

Additionally, *S. camelicanis* infection in the esophagus increased the IL-6 expression from 0.66 ± 0.05 in healthy camels from Riyadh to 2.67 ± 0.09 in infected camels from Riyadh (Figure 2D). In addition, IL-6 expression increased from 1.57 ± 0.50 in healthy animals to 4.71 ± 1.04 in animals infected with *S. camelicanis* (Figure 2D). There were no differences that could be attributed to the sex of these animals.

## Discussion

Animals infection with parasites is considered a problem for breeders, veterinarians and researchers. *Sarcocystis* spp. are one of the most frequent parasites known to infect animals, reptiles, and birds [5,33]. *Sarcocystis* infection is known to be a key factor for severe inflammatory diseases such as hepatitis, intestinal infection, encephalitis, and encephalomyelitis [34,35]. *Sarcocystis* can be rendered non-infectious by cooking meat at high temperature (100°C) for 5 min or freezing at low temperature (−20°C) for at least 2 days [33]. The aim of the present study was to investigate the potential effects of *Sarcocystis* spp. infection on inflammation in naturally infected Arabian camels.

We used the pepsin digestion method to detect thick walled Sarcocystis cysts, although no bradyzoites were found; this may be because the digestion time was not sufficient to digest the cyst or because the cysts were resistant to the digestion solution. The results of the present study do not report any myositis foci, which may be related to chronic infection in all cases, and therefore correlation to IL-6 expression is needed.

Our results described the architecture and structural ornaments of the Pcw and the protrusions arising from it. These criteria are used to identify the different species of *Sarcocystis* by majority of researchers [36–39]. The Flp of Pcw identified in the present study may be similar to some other identified species; however, the Kls, which arise from the Pcw in each protrusion, are a robust characteristic feature. Only mature cysts are used for identifying the species [39–43]. The morphology of *S. camel* observed in the present study conforms to the TEM classification by Dubey et al. [23]. The results obtained in the present study are similar to those obtained by [44], who studied *Sarcocystis* infection in camels. However, they described cone-like protrusions of the Pcw instead of Flp with Kls as shown here [44]. Currently, TEM analyses of *Sarcocystis* cysts now also need to be accompanied by molecular examination.

Our study clearly demonstrated that *Sarcocystis* infection increased inflammation by inducing IL-6 expression in different organs of camels, especially camels from the Al-Qassim Region. This may be due to the two populations of camels having different populations of microcysts in their tissues or to one of the places (Al Qassim) having animals with degenerating cysts. The tissue response to vital cysts is minimal, whereas the immune reaction to degenerating cysts is severe and causes tissue damage. No studies have investigated IL-6 expression in camels infected with Sarcocystis to compare with our study.

Recently, it was documented that the Arab camel virus or other parasitic infections can cause many inflammatory diseases that are transmissible to humans. This includes Middle East Respiratory Syndrome (MERS), The WHO (https://www.who.int/emergencies/mers-cov/en/) states that 858 MERS-CoV-associated deaths have occurred since 2012. Inflammatory symptoms previously associated with camel-borne disease may be caused by imbalances in the gut microbiome. We are implying that camel meat-associated diseases cause this microbiome imbalance. Thus, camel meat needs to be treated accordingly by freezing or cooking prior to being used as a food source.

Inflammation is a complex process that involves many pro- and anti-inflammatory cytokines and is a major feature of many diseases, including infectious diseases. A persistent state of inflammation is thought to favor tumorigenesis by stimulating angiogenesis [45]; this can produce chronic damage, leading to certain types of cancers [46–48] such as colorectal cancer [49,50] and can continuously stimulate chronic cell proliferation [51,52]. Organs, such as the tongue and tissue cells, such as gingival, lung, and esophageal cells, have been shown to produce a large array of cytokines including IL-6, which is known to be important for inflammation in these tissues [53,54]. As a proinflammatory cytokine with pleiotropic function, IL-6 is primarily involved in orchestrating and coordinating the innate and adaptive immune response via the STAT3 intracellular signaling pathway [55,56].

## Conclusions

Here, we used microscopy and RT-qPCR to investigate *Sarcocystis* spp. infection in *Camelus dromedaries* and to detect if there was variation in infection and immune response between different regional camel populations in Saudi Arabia. We identified *S. cameli* infection via light and TEM microscopy in camel tissue and organs and demonstrated that infection was more abundant in the Riyadh camel population compared with the Al-Qassim population. Expression of IL-6 was elevated in the majority of tissues compared with non-infected tissues, with large fold increases in infected tongue and cardiac muscle from the Al-Qassim population.

Zoonotic infections from infected animals, together with subsequent inflammatory reactions and chronic diseases are major burdens to global health, and this type of infection will likely worsen in the next 10 years in the Middle East. Research strategy programs should aim to address these critical challenges to our health and the global economy.

Our study indicates that regional variation in infection burdens in animal populations are likely and thus may affect subsequent human infections and their treatment.

## Data Availability

All data are available in the manuscript.

## Abbreviations

cDNA: complementary DNA
CRP: C-reactive protein
CT: cycle threshold
Flp: finger-like protrusions
IL: interleukin
Kls: knob-like structures
MERS: Middle East Respiratory Syndrome
qPCR: quantitative polymerase chain reaction
TEM: transmission electron microscopy
TLR: Toll-like receptor
